# FGF-21 and GDF-15 are increased in migraine and associated with the severity of migraine-related disability

**DOI:** 10.1186/s10194-023-01563-8

**Published:** 2023-03-20

**Authors:** Jiahui He, Mengting Zhou, Fanglin Zhao, Hongrong Cheng, Hao Huang, Xiaopei Xu, Jian Han, Wenwu Hong, Faming Wang, Yujin Xiao, Jinjin Xia, Kaiming Liu

**Affiliations:** 1 Department of Neurology, The Second Affiliated Hospital, Zhejiang University School of Medicine, No 88 Jiefang Road, Hangzhou, Zhejiang China; 2 Department of Anesthesiology, The Second Affiliated Hospital, Zhejiang University School of Medicine, No 88 Jiefang Road, Hangzhou, Zhejiang China; 3 Department of Radiology, The Second Affiliated Hospital, Zhejiang University School of Medicine, No 88 Jiefang Road, Hangzhou, Zhejiang China; 4 Department of Neurology, Affiliated Hospital of Shaoxing University, No 999 Zhongxingnan Road, Shaoxing, Zhejiang China; 5 Department of Neurology, Tiantai People’s Hospital of Zhejiang Province, No 1 Kangning Middle Road, Taizhou, Zhejiang China; 6 Zhejiang Chinese Medical University Affiliated Jiaxing TCM Hospital, 1501 East Zhongshan Road, Jiaxing, Zhejiang China; 7 Department of Neurology, Changxing People’s Hospital of Zhejiang Province, No 66 Taihu Middle Road, Changxing, Huzhou, Zhejiang China

**Keywords:** GDF-15, FGF-21, Migraine, Metabolism, Migraine-related disability

## Abstract

**Background:**

Migraine is a prevalent disorder with significant socioeconomic impact. The impairment of metabolic homeostasis in migraine warrants further investigation. Changes in serum levels of Fibroblast-growth-factor 21 (FGF-21) and Growth-differentiation-factor 15 (GDF-15) are characteristic of some metabolic and mitochondrial diseases. This study aimed to assess whether the presence of migraine affects serum levels of FGF-21 and GDF-15, and taking metabolic disorders into account as potential confounding factors.

**Methods:**

We collected serum samples from 221 migraine patients (153 episodic migraineurs and 68 chronic migraineurs) and 124 healthy controls. The serum concentrations of FGF-21 and GDF-15 were measured using an enzyme-linked immunosorbent assay (ELISA) based approach. Clinical variables, including monthly headache days, peak headache pain intensity, the 6-item Headache Impact Test (HIT-6), and the Migraine Disability Assessment (MIDAS), were also addressed. The associations between the clinical variables of migraine patients and serum levels of FGF-21 and GDF-15 were studied.

**Results:**

In the multiple regression that corrected for age, we found that the serum levels of FGF-21 and GDF-15 were significantly higher in migraine sufferers than in healthy controls. A significant elevation in serum concentration of FGF-21, but not GDF-15, was observed in patients with chronic migraine (CM) compared to those with episodic migraine (EM). Regarding migraine-related disability, higher scores on the HIT-6 and MIDAS were associated with higher levels of FGF-21 and GDF-15. For the receiver operating characteristic (ROC) analysis, the diagnosis of migraine using GDF-15 showed that the area under the ROC curve (AUC) was 0.801 and the AUC of chronic migraine was 0.880.

**Conclusion:**

Serum GDF-15 and FGF-21 levels are increased in patients with migraine and associated with the severity of migraine-related disability.

## Background

Migraine is a widespread neurological disorder that affects a substantial portion of the global population, with an estimated prevalence of over 15%. As a primary headache disorder that is debilitating in nature, it is ranked second in terms of the number of disability-adjusted life years it causes globally, and holds the top position among the causes of such years among young women [1]. The diagnosis of migraine is established through a comprehensive evaluation of the patient's clinical presentation, which includes an assessment of their medical history, headache duration, accompanying symptoms, and response to treatment. However, the diagnostic criteria have limitations as they do not fully capture the heterogeneity of migraine, including the potential role of metabolic abnormalities as contributing factors.

Evidence supports the idea that mitochondrial dysfunction and an imbalance between energy supply and demand may play a role in the pathophysiology and susceptibility to migraine [2]. Certain triggers, such as skipping meals or fasting, excessive exercise [3–5], dehydration, hypoxia [6–8], and lack of sleep [9], have been shown to have a clear link to metabolism. Intense sensory stimuli, including odors [10], phthalate-containing perfumes [11], blue light [12], and loud noises [13], can contribute to an increase in oxidative stress. Neuroimaging studies have revealed impairments in mitochondrial oxidative phosphorylation, reduced levels of ATP, and elevated lactate levels in the brains of patients with migraine [14, 15]. During a migraine attack, it has been observed that the brain's energy homeostasis is restored and harmful oxidative stress levels are reduced [2]. Studies have shown that oxidative mitochondrial metabolism is impaired in migraine patients including platelet mitochondrial dysfunction and production of peripheral markers of oxidative stress [16]. Okada et al. reported higher lactic acid levels in migraine patients [17], suggesting that mitochondrial dysfunction may be involved in the development of migraines. Animal models have also demonstrated that impairments in mitochondrial function can lead to the development of migraines, further supporting the role of mitochondrial dysfunction in migraine pathogenesis [18]. Furthermore, the activity of various mitochondrial enzymes, such as monoamine oxidase, succinate dehydrogenase, NADH dehydrogenase, cycloxygenase, and citrate synthetase, has been found to be decreased in the platelets of migraine patients with or without aura [19, 20]. Mitochondrial dysfunction may cause a decrease in energy production in the brain, leading to an increased susceptibility to migraine attacks [21]. Mitochondrial-targeted therapies have shown promising results in reducing the frequency and severity of migraine attacks, providing additional evidence for the role of mitochondrial impairment in migraines [22, 23].

Mitochondrial stress has been shown to impact the expression and secretion of fibroblast growth factor-21 (FGF-21) and growth differentiation factor-15 (GDF-15) [24]. GDF-15, a member of the transforming growth factor-β family, is known for its ability to regulate systemic energy metabolism by influencing appetite and food intake in the brainstem and hypothalamus. This protein is widely expressed in human tissues and has demonstrated neuroprotective effects in the brain [25–27]. Elevated blood levels of GDF-15 may be a reflection of mitochondrial function in patients [25] and have been shown to protect neuron cells from toxicity by preserving mitochondrial function and reducing apoptosis [28, 29]. FGF-21, which is primarily known as a hepatokine, regulates sugar intake through the central nervous system. Additionally, FGF-21 is involved in various cellular activities, including mitosis and viability, and is generally induced by mitochondrial-dependent mechanisms. Both FGF-21 and GDF-15 are considered to be circulating markers of mitochondrial disorders [30, 31] and have been linked to the severity of such diseases [29]. They are classified as stress-responsive cytokines that can modulate energy balance and play a role in the development of obesity and related comorbidities [32], as well as other diseases such as cancer, cardiovascular disease, diabetes, osteoporosis, and neurodegenerative disorders [30, 33].

It remains elusive for the role of FGF-21 and GDF-15 in migraine. Hence, in this study, we analyzed serum concentrations of FGF-21 and GDF-15 in patients with migraine and healthy controls and the relationship of these cytokines with patients’ clinical parameters.

## Materials and methods

### Participants

We evaluated the serum level of GDF-15 and FGF-21 in migraine patients and healthy controls from September 2019 to January 2023. The diagnosis of migraine is based on the criteria for migraine of International Classification of Headache Disorders 3rd edition criteria. According to attack frequency, patients were split into episodic migraine (EM) and chronic migraine (CM). Exclusion criteria were: (a) under the age of 18; (b) no informed consent could be taken; (c) at least one of the following criteria is met: proved or supposed mitochondrial disorder, cardiac diseases, cancer and chronic inflammatory diseases; (d) other primary or secondary headache disorders.

The local ethics committee of the second affiliated hospital of Zhejiang University approved the study. Written informed consent was obtained from all individual participants included in the study.

### Plasma Concentrations of FGF-21 and GDF-15

Plasma FGF-21 and GDF-15 were measures using enzyme-linked immunosorbent assay (ELISA) kits (Abcam, MA, USA). Blood samples were drawn from a peripheral vein into a 9 mL promoting coagulating tubes. Blood samples were centrifuged at 2500 g for 15 min and plasma was then transferred to polypropylene tubes and stored at − 80℃. The scientist who performed the assays was blinded to the study group the sample belonged to.

### Clinical assessment

Monthly headache days, peak headache pain intensity, concomitant symptoms, the presence of aura symptoms and the family history of headache were addressed. Furthermore, participants in the migraine group were asked to fill out Migraine Disability Assessment (MIDAS) and Headache Impact Test (HIT-6) to measure the degree of migraine-related functional disability. Patients Health Questionnaire (PHQ-9) and Generalized Anxiety Disorder (GAD-7) questionnaires were performed to measure the degree of migraine-related anxiety and depression.

### Statistical analysis

SPSS 23.0 statistics were used for statistics. Kolmogorov–Smirnov test was used to check the normality of the data distribution. Values were expressed as mean ± SE or median (interquartile range). Data were analyzed by one-way analysis of variance (ANOVA) followed by Dunnett’s post hoc test for multiple comparisons. Kruskal–Wallis test was used to evaluate the differences between CM and EM. Logistic regression analyses adjusted for age was used for groups comparison. Receiver operating characteristic (ROC) analysis explored the diagnostic ability of serum FGF-21 and GDF-15. Partial correlation analysis was conducted to detect the potential associations between cytokine levels and clinical parameters as well as questionnaires. The individual statistical tests are indicated within the results section. The *p* < 0.05 was considered statistically significant.

## Results

### Demographics

A total of 221 migraine patients (153 EM and 68 CM) and 124 healthy controls were recruited. In the CM group, 33 patients (48.53%) had comorbid medication-overuse headache (MOH). Patient demographics are summarized in Tables 1 and 2. The age and gender of migraineurs and normal controls were matched [age: 40.50 ± 0.88 (migraineur) vs. 38.98 ± 1.00 (control), *p* = 0.815; gender (male): 22.17% (migraineur) vs. 19.35% (control), *p* = 0.789, Table 1]. Patients with CM were older than EM [45.38 ± 1.77 (CM) vs. 38.33 ± 0.95 (EM), *p* < 0.001). Patients with CM exhibit not only more headache frequency but also more severe symptoms than patients with EM [monthly headache days, *p* < 0.001; peak headache pain intensity, *p* = 0.041].

**Table 1:** Characteristics of migraine patients and healthy controls. Values are presented as mean ± SE

Characteristics	Control Group (*n* = 124)	All Migraine (*n* = 221)	CM (*n* = 68)	EM (*n* = 153)
age, years	38.98 ± 1.00	40.50 ± 0.88	45.38 ± 1.77	38.33 ± 0.95
BMI, kg/m2	21.92 ± 0.29	22.11 ± 0.24	22.48 ± 0.40	21.95 ± 0.30
males, n (%)	24 (19.35%)	49 (22.17%)	9 (13.24%)	40 (26.14%)
FGF-21, pg/mL	103.49 ± 9.36	259.53 ± 18.56	313.68 ± 38.33	235.30 ± 20.47
GDF-15, pg/mL	401.63 ± 19.33	944.44 ± 35.55	1022.45 ± 55.06	909.76 ± 44.97

*CM *Chronic migraine, *EM *Episodic migraine

**Table 2:** Characteristics of CM and EM patients. Average headache days were calculated in the previous three months. Headache intensity was evaluated using VAS. For female patients, the relationship between headache attack and menstruation was counted. Values are presented as mean ± SE. Kruskal–Wallis test was used to analysis the difference of clinical parameters between CM and EM. The mean values were considered different if *p* < 0.05

Clinical parameters	CM (*n* = 68)	EM (*n* = 153)	*p*-Value
age, years	45.38 ± 1.77	38.33 ± 0.95	< 0.001
BMI, kg/m2	22.48 ± 0.40	21.95 ± 0.30	0.217
males, n (%)	9 (13.24%)	40 (26.14%)	0.168
monthly headache days	23.12 ± 0.85	3.73 ± 0.23	< 0.001
peak headache pain intensity (VAS)	6.53 ± 0.10	6.31 ± 0.18	0.041
family history	41 positive /27 negative	113 positive/40 negative	0.068
migraine with aura, n (%)	9 (13.24%)	21 (13.73%)	0.937
nausea or vomit, n (%)	44 (64.71%)	115 (75.16%)	0.070
vertigo n (%)	26 (38.24%)	58 (37.91%)	0.988
photophobia and phonophobia, n (%)	37 (54.41%)	109 (71.24%)	0.028
menstrual migraine, n (%)	24 (40.68%)	63 (54.78%)	0.445
MOH, n (%)	33 (48.53%)	0	< 0.001
use of analgesics, n (%)	52 (76.47%)	47 (30.72%)	< 0.001
migraine-specific	9 (13.24%)	6 (3.92%)	0.008
non-migraine specific	48 (70.59%)	44 (28.76%%)	< 0.001
migraine preventive medication use, n (%)	61 (89.71%)	72 (47.06%)	< 0.001
antidepressant	7 (10.29%)	12 (7.84%)	0.657
antiepileptic	14 (20.59%)	31 (20.26%)	0.130
beta-blocker	6 (8.82%)	9 (5.88%)	0.545
calcium channel blocker	28 (41.18%)	48 (31.37%)	< 0.001
drug acting on the CGRP pathway	0	0	/
lisinopril or candesartant	1 (1.47%)	0	0.657
onabotulinumtoxin A	4 (5.88%)	0	0.011
other (e.g. Chinese traditional medicine)	12 (17.65%)	39 (25.49%)	0.407

*CM *Chronic migraine, *EM *Episodic migraine, *MOH *Medication-overuse headache, *VAS *Visual analogue scale

### FGF-21 and GDF-15 values between groups

**Fig. 1:**
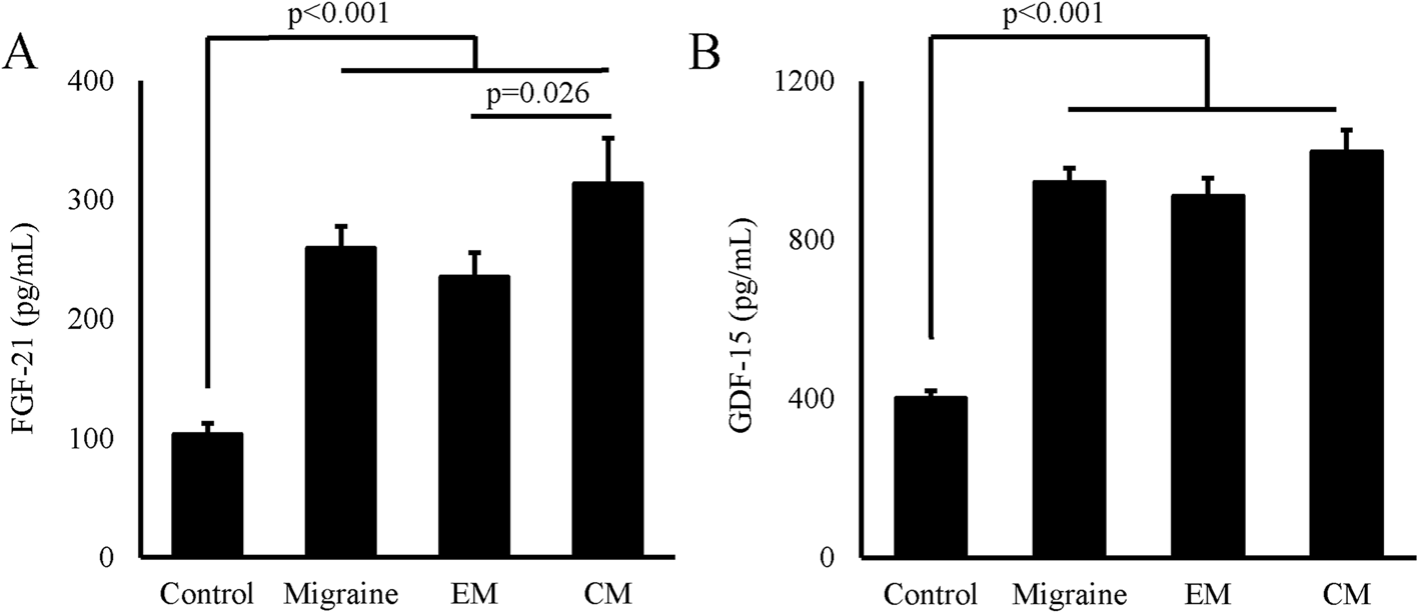
FGF-21 and GDF-15 values within the different groups. The *p* < 0.05 was considered statistically significant. CM: Chronic migraine; EM: Episodic migraine

In order to account for the influence of confounding variables on FGF-21 and GDF-15 concentrations, we took into consideration the factors of age in our analysis. Serum levels of both FGF-21 and GDF-15 in migraine patients were significantly higher than that in healthy controls (*p* < 0.001). Meanwhile, the concentration of FGF-21 in patients with CM were significantly higher than EM (*p* = 0.026) (Fig. 1). No difference between chronic and episodic migraine for GDF-15 was found in this study (*p* = 0.290). Furthermore, no significant difference in the concentrations of FGF-21 and GDF-15 was observed between the CM group with MOH and the non-MOH group [FGF-21: 272.26 ± 47.63 (MOH) vs. 274.06 ± 52.83 (non-MOH), *p* = 0.977; GDF-15: 1033.35 ± 89.12 (MOH) vs. 1016.87 ± 115.17 (non-MOH), *p* = 0.552].

### Receiver operating characteristic (ROC) curve analysis

**Fig. 2:**
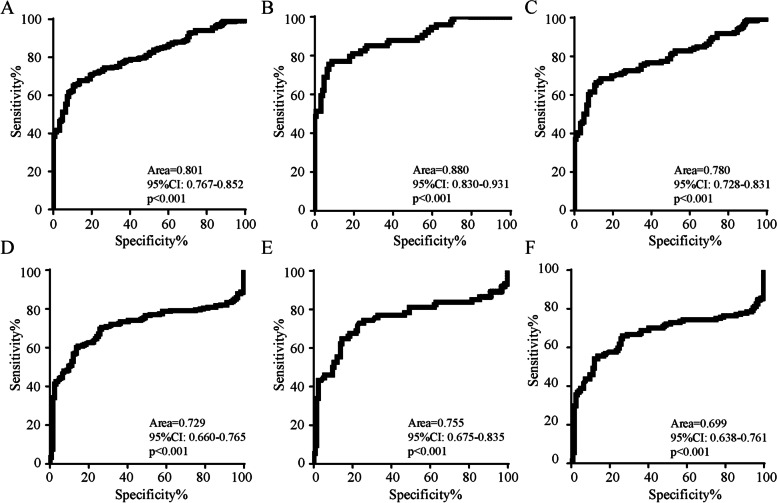
ROC curve analysis of serum GDF-15 for diagnosis of (**A**) migraine, **B** CM, (**C**) EM from control group. ROC curve analysis of serum FGF-21 for diagnosis of (**D**) migraine, **E** CM, **F** EM from control group. CM: Chronic migraine; EM: Episodic migraine; ROC: Receiver operating characteristic

We calculated the area under the receiver operating characteristic (ROC) curve to evaluate the effectiveness of FGF-21 and GDF-15 prediction model for patients with migraine (Fig. 2). The area under curve (AUC) in predicting migraine, EM, CM in all subjects for GDF-15 was 0.801 (sensitivity 64.62%; specificity 89.66%), 0.780 (sensitivity 65.32%, specificity 86.29%), 0.880 (sensitivity 75.00%, specificity 92.00%), respectively. For serum FGF-21, the AUC in predicting migraine, EM, CM in all subjects for FGF-21 was 0.729 (sensitivity 59.70%; specificity 87.93%), 0.755 (sensitivity 59.06%, specificity 86.29%), 0.755 (sensitivity 65.79%; specificity 85.60%), respectively.

### Relationship between serum factors and clinical variables

**Fig. 3:**
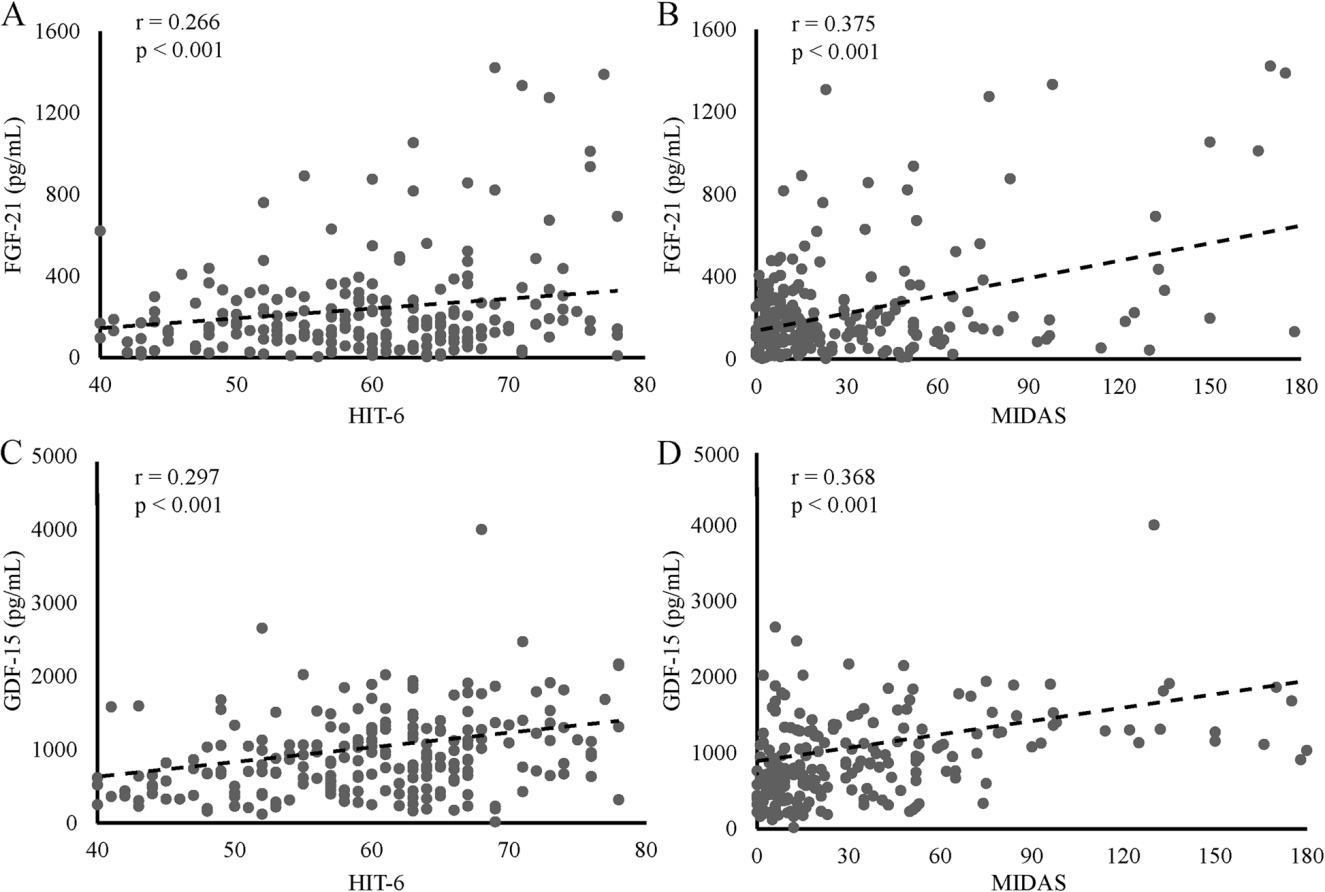
Correlation between cytokines and clinical variables in migraineurs. **A** FGF-21 and HIT-6. **B** FGF-21 and MIDAS. **C** GDF-15 and HIT-6. **D** GDF-15 and MIDAS. HIT-6: 6-item Headache Impact Test; MIDAS: Migraine Disability Assessment

In migraineurs, the correlation of FGF-21 and GDF-15 with each clinical parameter are summarized in Table 3. We found that FGF-21 and GDF-15 exhibited a positive association with aging. There was no correlation of serum FGF-21 and GDF-15 in migraine with or without aura, nausea or vomit, vertigo and menses (Table 3). We performed a correlation analysis between the cytokines and the burden of migraine, as well as anxiety and depression (Fig. 3). Accelerated disability according to HIT-6 and MIDAS was associated with higher serum level of FGF-21 (HIT-6: r = 0.266, *p* < 0.001, MIDAS: r = 0.375, *p* < 0.001) and GDF-15 (HIT-6: r = 0.297, *p* < 0.001,MIDAS: r = 0.368, *p* < 0.001). There was no statistical correlation between PHQ-9 and GAD-7 and cytokines.

**Table 3:** Clinical parameters of headache and their impact on FGF-21 and GDF-15 levels in migraineurs. Partial correlation analysis was used for analysis. The results are given as r values (*p* values). The results are given as *p* values

Clinical parameters	FGF-21	GDF-15
age	0.285 (*p* < 0.001)	0.233 (*p* < 0.001)
BMI	0.075 (*p* = 0.254)	-0.083 (*p* = 0.204)
gender	0.088 (*p* = 0.179)	0.060 (*p* = 0.356)
monthly headache days	0.061 (*p* = 0.357)	0.017 (*p* = 0.799)
peak headache pain intensity (VAS)	-0.065 (*p* = 0.328)	0.096 (*p* = 0.146)
family history	-0.148 (*p* = 0.035)	-0.004 (*p* = 0.960)
migraine with aura	-0.007 (*p* = 0.918)	0.037 (*p* = 0.602)
nausea or vomit	-0.076 (*p* = 0.247)	-0.107 (*p* = 0.102)
vertigo	-0.022 (*p* = 0.756)	0.001 (*p* = 0.993)
photophobia and phonophobia	-0.005 (*p* = 0.938)	-0.111 (*p* = 0.112)
MOH	-0.037 (*p* = 0.623)	0.093 (*p* = 0.213)
menstrual migraine	-0.084 (*p* = 0.294)	0.041 (*p* = 0.609)

*MOH *Medication-overuse headache, *VAS *Visual analogue scale

## Discussion

This study investigated whether the presence of migraine affects serum levels of FGF-21 and GDF-15, both of which have been implicated in metabolic disorders. An increasing number of studies have indicated the impairment in mitochondrial homeostasis has been proposed as a potential contributor to the pathophysiology and susceptibility of migraine [34–37]. FGF-21 and GDF-15 are considered to be markers of mitochondrial disorders [24, 25] and have been linked to the severity of such diseases [23]. Despite all this, the levels of FGF-21 and GDF-15 are likely influenced by age, body max index (BMI) and some certain diseases (e.g. heart failure, myocardial infarction, pulmonary hypertension, diabetes, metabolic syndrome, autoimmune diseases, and cancer) [38, 39]. Since these specific diseases have been listed as exclusion criteria, the remaining confounding variables in this research mainly include gender, BMI, and age. It was confirmed that age, BMI, and gender were matched when comparing the migraine and control groups. The study found significantly heightened serum levels of FGF-21 and GDF-15 in patients with migraine compared to a control group, which suggests that both cytokines may have potential value in distinguishing between migraine sufferers and healthy controls.

In this study, the migraine group comprised approximately 30% of individuals with CM, almost half of whom reported experiencing MOH. In comparison of CM and EM, the BMI and gender were matched between the two groups, but age was not, the individuals with CM were older than those with EM. CM is known to develop from EM [40, 41], and the mean age at onset of CM was higher than that of EM [42, 43]. Importantly, age had a significant effect on GDF-15 and FGF-21 levels. As blood indexes closely related to mitochondrial stress, FGF-21 and GDF-15 were established to be related to aging and age-related diseases [44]. Our research found a positive correlation between age and the concentrations of GDF-15 and FGF-21 not only in the migraine group but also in the healthy group. Given that the observed difference in cytokines might be influenced by the older age of CM patients, we corrected for age in the multiple regression when comparing the levels of cytokines in CM and EM patients, however, the significant difference in FGF-21 between the two groups persisted.

Additionally, among the migraine group, higher concentrations of FGF-21 and GDF-15 were significantly correlated with increased migraine-related burden and disability, as evidenced by higher scores on the HIT-6 and MIDAS assessments. The higher concentrations of serum FGF-21 and GDF-15 observed in individuals with migraine and increased burden and disability suggest a potential correlation between disease severity. Analogous circumstances are also encountered in other disease. A recent meta-analysis of individual patients showed that GDF-15 demonstrated a strong and consistent independent association with cardiovascular death and heart failure across all presentations of atherosclerotic cardiovascular disease [45]. In a longitudinally sampled cohort of patients with multiple sclerosis, mean GDF-15 concentrations may serve as a biomarker for disease stability [46].

Furthermore, there have been several randomized control trials to evaluate the efficacy of metabolic-targeted nutraceuticals such as coenzyme Q10, magnesium, and riboflavin in the prevention of migraine. These trials have shown promising results in reducing the frequency and severity of migraine attacks, and it is recommended by migraine treatment guidelines in multiple countries [47]. The question of whether the levels of FGF-21 and GDF-15 can serve as a reference for the personalized use of nutraceuticals in the treatment of migraine is a topic that merits further investigation.

This study is still subject to limitations. Our results are based on cross-sectional observation, and longitudinal data is necessary to validate our conclusions. Furthermore, a larger study with a more detailed assessment would be necessary to confirm these relationships. Life circumstances, such as food habits or physical activity, were not considered. In addition, serum samples were obtained from patients, regardless of whether they were experiencing a migraine attack or were in a period of remission. There are significant differences in medication use between CM and EM patients, including analgesics and preventive medications. Due to the extremely complex types and dosages of these medications, it is difficult to include all of them as corrective factors in our analysis of the results, which may have introduced bias into the analysis of our results. While as an additional investigation, we conducted a comparative analysis of the concentrations of FGF-21 and GDF-15 in the MOH and non-MOH subgroups of CM patients, and did not find a significant difference of excessive use of analgesics on metabolic levels. This research is deficient in mitochondria-associated parameters as measured in the serum of patients, especially those that are downstream to FGF-21 and GDF-15. The elevated levels of GDF-15 and FGF-21 in patients with migraine are insufficient to support the potential mechanism of mitochondria in migraine.

We have demonstrated that serum levels of GDF-15 and FGF-21 are elevated in patients with migraine. The heightened concentrations of GDF-15 and FGF-21 are linked to greater disease burden, indicating their potential as peripheral blood markers for evaluating the severity of migraine-related disability.

## Data Availability

Datasets are available on request: The raw data supporting the conclusions of this article will be made available by the authors, without undue reservation.

## Abbreviations

ANOVA: Analysis of variance
AUC: Area under the curve
BMI: Body mass index
CM: Chronic migraine
ELISA: Enzyme-linked immunosorbent assay
EM: Episodic migraine
FGF-21: Fibroblast growth factor-21
GAD-7: Generalized Anxiety Disorder
GDF-15: Growth differentiation factor-15
HIT-6: 6-Item Headache Impact Test
MIDAS: Migraine Disability Assessment
MOH: Medication-overuse headache
PHQ-9: Patients Health Questionnaire
ROC: Receiver operating characteristic
